# *BCL10* is rarely mutated in human prostate carcinoma, small-cell lung cancer, head and neck tumours, renal carcinoma and sarcomas

**DOI:** 10.1038/sj.bjc.6690561

**Published:** 1999-07

**Authors:** S Gill, J Broni, S Jefferies, P Osin, G Kovacs, N J Maitland, R Eeles, S M Edwards, M J S Dyer, T G Willis, the M. P. T. Collaborators, the St George's Hospital Collaborators, C S Cooper

**Affiliations:** Sections of Molecular Carcinogenesis, Cancer Genetics, Cell Biology and Experimental Pathology, Academic Haematology, Institute of Cancer Research, 15 Cotswold Road, Sutton, Surrey SM2 5NG, UK; Molecular Oncology, Department of Urology, University of Heidelberg, Im Neuenheimer Feld, 365, 69120 Heidelberg, Germany; YRC Cancer Research Unit, University of York, Heslington, York, YO10 5YW, UK.; See acknowledgements

**Keywords:** *BCL10*, mutation, prostate cancer, sarcomas, lung cancer, renal carcinoma, head and neck squamous cell cancers

## Abstract

We have used single-strand conformation polymorphism (SSCP) analysis to screen for mutations in the *BCL10* gene in 81 primary prostate carcinomas, 20 squamous cell cancers of the head and neck, 15 small-cell lung cancer cell lines, 24 renal carcinoma cell lines and 13 sarcoma cell lines. We failed to find evidence of somatically acquired mutations of the *BCL10* gene suggesting that *BCL10* does not play a major role in the development of these malignancies. © 1999 Cancer Research Campaign


British Journal of Cancer (1999) **80**(10), 1565–1568
© 1999 Cancer Research Campaign
Article no. bjoc.1999.0561

Short Communication

### *BCL10* is rarely mutated in human prostate carcinoma, small-cell lung cancer, head and neck tumours, renal carcinoma and sarcomas

**S Gill[1], J Broni[1], S Jefferies[2], P Osin[3], G Kovacs[4], NJ Maitland[5], R Eeles[2], SM Edwards[2], MJS Dyer[6], TG Willis[6], the MPT Collaborators[7], the St George's Hospital Collaborators[7] and CS Cooper[1]**

Sections of [1]Molecular Carcinogenesis, [2]Cancer Genetics, [3]Cell Biology and Experimental Pathology, [6]Academic Haematology, Institute of Cancer Research, 15 Cotswold Road, Sutton, Surrey SM2 5NG, UK; [4]Molecular Oncology, Department of Urology, University of Heidelberg, Im Neuenheimer Feld, 365, 69120 Heidelberg, Germany; [5]YRC Cancer Research Unit, University of York, Heslington, York, YO10 5YW, UK. [7]See acknowledgements.

**Summary** We have used single-strand conformation polymorphism (SSCP) analysis to screen for mutations in the *BCL10* gene in 81 primary prostate carcinomas, 20 squamous cell cancers of the head and neck, 15 small-cell lung cancer cell lines, 24 renal carcinoma cell lines and 13 sarcoma cell lines. We failed to find evidence of somatically acquired mutations of the *BCL10* gene suggesting that *BCL10* does not play a major role in the development of these malignancies.

**Keywords:** *BCL10*; mutation; prostate cancer; sarcomas; lung cancer; renal carcinoma; head and neck squamous cell cancers

The *BCL10* gene (*CIPER*, Koseki et al, 1999; *c-CARMEN*, Thome et al, 1999; *mE10*, Yan et al, 1999) was originally identified as the gene that becomes fused to the *IgH* locus in MALT B-cell lymphomas as a consequence of the t(1;14)(p22;q32) translocation and is a cellular homologue of the equine herpes virus-2 E10 gene (Willis et al, 1999; Zhang et al, 1999). Both encode proteins that contain an amino terminal caspase recruitment domain (CARD) similar to that found in some apoptotic regulatory proteins (Hoffman et al, 1997). The *BCL10* gene in lymphomas with the t(1;14) translocation was altered by frameshift mutations that resulted in truncation of the BCL10 protein beyond the CARD domain. It was suggested that these mutations might arise as a consequence of the *Ig* gene somatic hypermutation mechanism (Migliazza et al, 1995; Matolcsy et al, 1996). Mutations resulting in similarly truncated proteins were also reported in cDNA clones from 3/3 mesothelioma and 3/3 germ cell tumour cell lines, and in genomic DNA from 2/25 colorectal adenocarcinomas. In contrast, mutation was not observed in 15 breast carcinomas, 11 pancreatic adenocarcinomas or 15 lung carcinoma cell lines. Willis et al (1999) also showed that wild-type BCL10 protein could induce apoptosis; however, the truncated BCL10 protein failed to induce apoptosis and exhibited transforming activity. In the current study we have extended the investigation of the occurrence of *BCL10* mutations to other tumour types, including prostate carcinoma, small-cell lung cancers, head and neck tumours, kidney cancer and sarcomas.

### METHODS

### Tumour samples and cell lines

Prostate tissue from patients undergoing either transurethral resection of prostate or radical prostatectomy, was either immediately frozen (71/87 of the samples) or preserved by formalin fixation (16/87 of the samples). Microdissection of frozen material was carried out as described previously (Macintosh et al, 1998) on haematoxylin and eosin (H&E) stained sections using a glass needle. Alternatively, where larger areas of tumour were apparent, microdissection was carried out by hand using a scalpel. Microdissection of paraffin-embedded material was carried out on five parallel 10 μm sections stained with toluidine blue. Foci of carcinoma were excised and the histology examined in an adjacent 5 μm section stained with H&E. All prostate carcinomas were graded G1–4 according to D'Amico et al (1998). The grading pattern for this set of samples was G0 6/87 (7%), G1 4/87 (5%), G2 43/87 (49%), G3 14/87 (16%), G4 14/87 (16%), ungraded 6/87 (7%).

The two prostate carcinoma cell lines examined were LNCaP and PC3. Fifteen small-cell lung cancer cell lines were examined HTB119, HTB120, HTB171, HTB173, HTB175, HTB180, HTB184, CRL 2049, CRL 2062, CRL 2064, CRL 2066, CRL 5804, CRL 5808, CRL 5809 and CRL 5811. Eighteen clear cell renal carcinoma cell lines were examined UOK101, 102, 108, 111, 122, 125, 126, 138, 139, 140, 141, 147, 151, 154, 161, 170, 171 and 180. Six papillary renal cell carcinoma cell lines were examined UOK109, 120, 124, 145, 112 and 132. The first four of these are known to contain translocations involving the *TFE3* gene (Sidhar et al, 1996; Clark et al, 1997). Eleven sarcoma cell lines examined were STS255, Fuji, HS-SY-II and A2243 synovial sarcomas; A204, RMS and A673 rhabdomyosarcomas; MNNG-HOS chemically transformed human osteosarcoma line; HT1080 and HTB152 fibrosarcomas; RD-ES and HTB86 Ewings sarcomas and the HTB88 leiomyosarcoma. The SK-N-BE and TN2 neuroblastoma cell lines, A431 epidermoid carcinoma cell line, the SK23 melanoma cell line and BT474 breast carcinoma cell lines were also examined.

A series of 20 head and neck squamous cell cancers consisting of index tumours of various sites from 16 patients and four second tumours that had developed within the head and neck region (more than 3 years from the original diagnosis) were also screened.

*Received 21 April 1999*
*Accepted 23 April 1999*

*Correspondence to:* S Gill

These tumours were graded G1–3 as follows: G1 4/20; G2 8/20; G3 4/20; ungraded 4/20. DNA was extracted from archival material.

Cell lines were from the American Tissue Culture Collection (ATCC), except for the UOK lines (kindly provided by Dr WM Linehan) (Anglard et al, 1992), RMS (Garvin et al, 1986), Fuji (Nojima et al, 1990), A2243 (kindly provided by Dr SA Aaronson), HS-SY-II (Sonobe et al, 1992) and TN2 (Yaoita et al, 1989) DNA was extracted from microdissected material and cell lines by established methods (Sambrook et al, 1989).

### SSCP analysis

Exons 1, 2 and 3 of *BCL10* were amplified by polymerase chain reaction (PCR) from genomic DNA using Hotstar *Taq* DNA polymerase (Quiagen) and the five primer sets (1, 2.1, 2.2, 3.1 and 3.2) described by Willis et al (1999). Following an initial incubation at 95°C for 15 min for enzyme activation the cycle conditions for the first five cycles were as follows: 94°C for 30 s, 65–55°C for 30 s (decreasing by 2.5°C per cycle) and 72°C for 1 min. The subsequent 35 cycles were 94°C 1 min, 55°C 1 min and 72°C 1 min, followed by a final step of 72°C for 5 min. For single-strand conformation polymorphism (SSCP) analysis PCR products (2 µl) were mixed with 4 µl formamide loading buffer and separated in the Genphor electrophoresis system (Pharmacia-Amersham, UK) under 15 W constant power for 2–3 h at 5°C. Bands were visualized by silver staining. DNA from the follicular lymphoma sample, G0109, was kindly provided by MJS Dyer for use as a positive control with exon 3.2 SSCP gels.

### Direct sequencing

Abnormal bands were excised from SSCP gels and heated in 50 µl water at 95°C for 10 min. One microlitre of eluate was used to reamplify the mutant product using the same primers and conditions. Reamplified products were excised from agarose gels, purified using a gel extraction kit (Quiagen) to remove primer dimers and excess nucleotides and sequenced in both directions using an ABI Prism Dye Terminator Cycle Sequencing Kit containing Amplitaq DNA polymerase FS (Perkin-Elmer) according to manufacturer’s instructions.

### RESULTS

In this study we have examined 81 prostate primary prostate carcinomas as well as the LNCaP and PC3 prostate carcinoma cell lines. Primary prostate carcinomas were microdissected from frozen or formalin-fixed specimens prior to genetic analysis in order to minimize contamination from normal tissue. Six specimens of benign prostatic hyperplasia were added to this series. Fifteen small-cell lung carcinoma cell lines, 24 renal cell carcinoma cell lines, 20 squamous cell carcinomas of the head and neck and 13 soft tissue sarcoma cell lines were also examined. Also included in this series were cell lines from two neuroblastomas, an epidermal carcinoma, a melanoma and a breast carcinoma.

To screen for mutations in the *BCL10* gene we have adopted the same PCR-SSCP based method utilized by Willis et al (1999). The full coding sequence of the *BCL10* gene was amplified by five different PCR reactions with a single PCR product spanning exon 1 and two reactions spanning each of the remaining two exons. This technique can detect mutations down to a sensitivity of

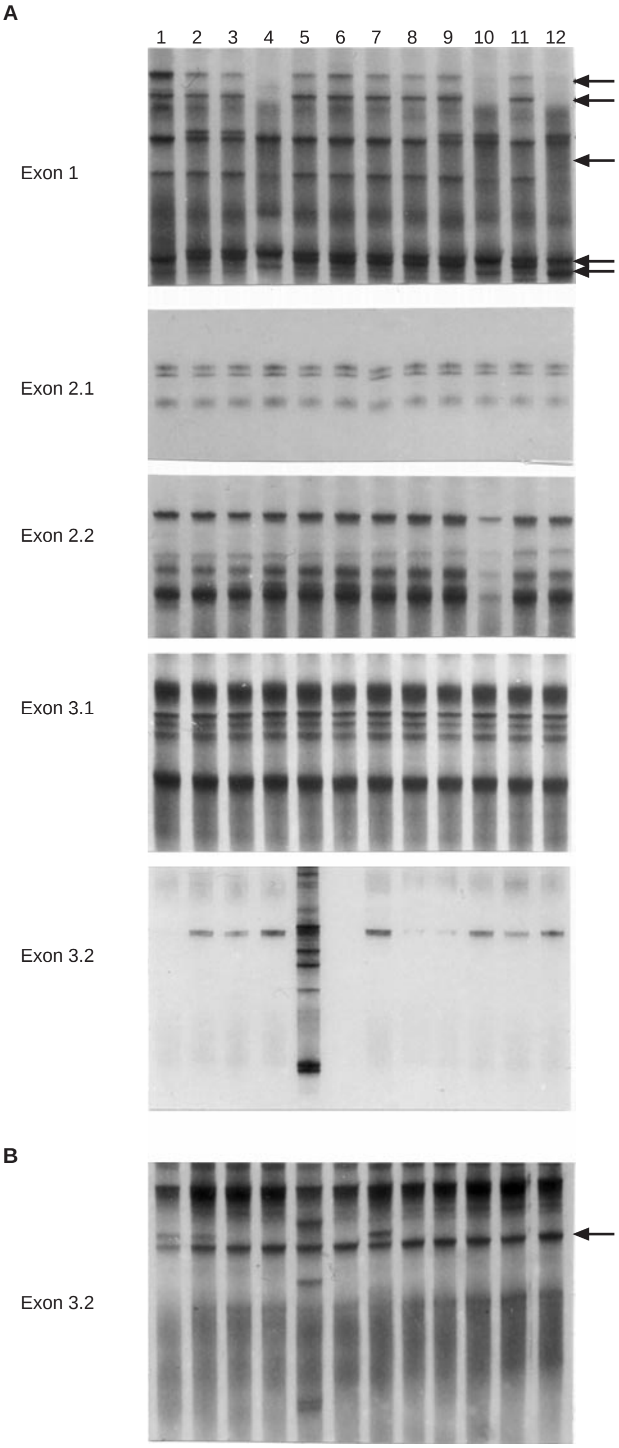

**Figure 1** (**A**) Examples of silver stained ‘Genophor’ SSCP gels of 12 prostate carcinoma specimens for each of the five *BCL10* exon fragments. Arrows denote bandshifts corresponding to polymorphic loci. Lane 5 of exon 3.2 contains the follicular lymphoma sample G0109 which harbours a deletion in exon 3.2 (Willis et al, 1999). For exon 3.2, lane 1 shows a failed PCR reaction, whilst lane 6 is a negative (water) control. (**B**) Silver-stained SSCP gels of exon 3.2 samples showing bandshifts attributed to a polymorphism. Lane 1 normal lymphocyte sample 1; lane 2 A431; lane 3 A204; lane 4 A2243; lane 5 G0109 as per (**A**); lane 6 HS-SY-II; lane 7 Fuji; lane 8 STS255; lane 9 UOK122; lane 10 UOK161; lane 11 UOK154; lane 12 normal lymphocyte sample 2

*© 1999 Cancer Research Campaign*

one mutant allele in ten normal alleles and other screening projects in our laboratory have yielded detection rates of 90% (Condie et al, 1993).

Analysis of exon 1 identified several abnormally migrating bands in SSCP gels. Each abnormal band was, however, present in many samples indicating that they represent polymorphisms rather than true mutations (Figure 1A). Sequencing all of the abnormal bands demonstrated that the alterations correspond to a silent CTG-CTC mutation at codon 8 and a G→C alteration 11 bases downstream from exon 1 within intron 1. These were either found alone or in combination. SSCP analysis of exon 2 and of the 5′ region of exon 3 failed to yield abnormally migrating bands. Finally, analysis of the 3′-region of exon 3 yielded a single abnormal band, which again was present in many samples (Figure 1B). This band was sequenced and found to correspond to a previously characterized GGA(gly)→GAA(glu) polymorphism at codon 213 (TG Willis, personal communication). Thus we failed to detect somatic mutations of *BCL10* in the tumour series examined in this study.

### DISCUSSION

It is now generally accepted that cancer development is a multistep process requiring the accumulation of mutations in several distinct genes. Alterations in these genes may have a variety of biological consequences, including constitutive activation of cellular control pathways, removal of cell cycle check points, removal of mechanisms which monitor DNA damage and interference with mechanisms that control entry into the apoptotic pathway (Lane, 1992; Vogelstein and Kinzler, 1993; Kinzler and Vogelstein, 1997). Mutations in the *BCL10* gene are believed to fall within the latter category of alterations and represented the first example of alterations of a protein containing a CARD domain in human cancer development. Willis et al (1999) found that mutations within the *BCL10* gene in human malignancy resulted in truncated BCL10 proteins that were no longer able to promote entry into apoptosis.

Willis et al (1999) reported a high frequency of *BCL10* mutations, in particular groups of malignancies including MALT lymphomas, follicular lymphomas, germ cell tumours and mesotheliomas, but much lower levels of mutations in other tumour classes such as lung cancer and colorectal cancer. The results presented in the current study are in agreement with these observations since we failed to detect alterations of the *BCL10* gene in small-cell lung cancer. In addition we have identified other groups of malignancies including clear cell and papillary renal carcinoma, prostate cancer and human sarcomas that also exhibit low frequencies of *BCL10* mutations. A limitation of the current study is that the SSCP may miss some mutations thus the occurrence of a low level of mutation within these tumour groups cannot be excluded. When the data are considered together it would, therefore, appear that alterations of *BCL10* may be restricted to particular classes of human malignancy. Additional studies are required on other types of cancer to determine the exact distribution of *BCL10* mutations in neoplasia.

### ACKNOWLEDGEMENTS

We would like to thank the Cancer Research Campaign for funding this work and Christine Bell for typing this manuscript. NJM is supported by Yorkshire Cancer Research. PO is supported by the 'Breakthrough' breast cancer charity. MPT Collaborators: S Jefferies[1], R Eeles[1], J Henk[2], M Gore[2], P Rhys-Evans[2], D Archer[2], K Bishop[2], E Solomon[3], S Hodgson[3], M McGurk[3], J Hibbert[3], M O'Connell[3], M Saunders[4], M Partridge[3], E Chevretton[3], F Calman[3], K Shotton[5], A Brown[6], S Whittaker[7]. [1]Cancer Genetics, Institute of Cancer Research, 15 Cotswold Road, Sutton, Surrey, SM2 5NG, UK. [2]The Royal Marsden Hospital Trust, Downs Road, Sutton, Surrey SM2 5PT, UK. [3]Guys and St Thomas' and Kings Hospital Trusts, London, UK. [4]Mount Vernon Hospital Trust, Rickmansworth Road, Northwood, Middlesex HA6 2RN, UK. [5]Mid-Kent Oncology Centre, Hermitage Lane, Maidstone, Kent ME16 9QQ, UK. [6]Queen Victoria Hospital. [7]Royal Surrey County Hospital, Egerton Road, Guildford, Surrey GU2 5XX, UK. St Georges Hospital Collaborators: R Eeles[1], R Kirby[2], C Corbishley[2], W Dunsmuir[2]. [1]See 1 above. [2]Urology Unit, St Georges NHS Trust, Blackshaw Road, Tooting, London SW17 0QJ, UK.

### REFERENCES

Anglard P, Trahan E, Liu S, Latif F, Merino MJ, Lerman MI, Zbar B and Linehan WM (1992) Molecular and cellular characterisation of human renal cell carcinoma cell lines. *Cancer Res* **52**: 348–356

Clark J, Lu YJ, Sidhar SK, Parker C, Gill S, Smedley D, Hamoudi R, Linehan WM, Shipley J and Cooper CS (1997) Fusion of splicing factor genes PSF and NonO (p54nrb) to the TFE3 gene in papillary renal cell carcinoma. *Oncogene* **15**: 2233–2239

Condie A, Eeles R, Borreson AL, Coles C, Cooper C and Prosser J (1993) Detection of point mutations in the p53 gene: comparison of single-strand conformation polymorphism, constant denaturant gel electrophoresis and hydroxylamine and osmium tetroxide techniques. *Hum Mutat* **2**: 58–66

D'Amico AV, Whittington R, Malkowicz B, Schultz D, Blank K, Broderick GA, Tomaszewski JE, Renshaw AA, Kaplan I, Beard CJ and Wein A (1998) Biochemical outcome after radical prostatectomy, external beam radiation therapy or interstitial radiation therapy for clinically localised prostate cancer. *JAMA* **280**: 969–974

Garvin AJ, Stanley WS, Bennett DD, Sullivan JL and Sens DA (1986) The in vitro growth, heterotransplanation and differentiation of a human rhabdomyosarcoma cell line. *Am J Pathol* **125**: 208–217

Hoffman K, Bucher P and Tschopp J (1997) The CARD domain: a new apoptotic signalling motif. *Trends Biochem Sci* **22**: 155–156

Kinzler KW and Vogelstein B (1997) Cancer-susceptibility genes. Gatekeepers and caretakers. *Nature* **386**: 761

Koseki T, Inohara N, Chen S, Carrio R, Merino J, Hottiger MO, Nabel GJ and Nunez G (1999) CIPER, a novel NF κB-activating protein containing a caspase recruitment domain with homology to *Herpesvirus*-2 protein E10. *J Biol Chem* **274**: 9955–9961

Lane DP (1992) p53, guardian of the genome. *Nature* **358**: 15–16

Macintosh CA, Stower M, Reid N and Maitland NJ (1998) Precise microdissection of human prostate cancer reveals genetic heterogeneity. *Cancer Res* **58**: 23–28

Matolcsy A, Casali P, Warnke RA and Knowles DM (1996) Morphologic transformation of follicular lymphoma is associated with somatic mutation of the translocated BCL-2 gene. *Blood* **88**: 3937–3944

Migliazza A, Martinotti S, Chen W, Fusco C, Ye BH, Knowles DM, Offit K, Chaganti RS and Dalla-Fevera R (1995) Frequent somatic hypermutation of the 5′ noncoding region of the BCL6 gene in B-cell lymphomas. *Proc Natl Acad Sci USA* **92**: 12520–12524

Nojima T, Wang Y-S, Abe S, Matsuna T, Yanawaki S and Nagashina K (1990) Morphological and cytogenetic studies of a human synovial sarcoma xenotransplanted into nude mice. *Acta Pathol Jap* **40**: 487–493

Sambrook J, Fritsch EF and Maniatis T (1989) Molecular cloning: a laboratory manual. Cold Spring Harbor Laboratory Press

Sidhar SK, Clark J, Gill S, Hamoudi R, Crew AJ, Gwilliam R, Ross M, Linehan WM, Birdsall S, Shipley J and Cooper CS (1996) The t(X;1)(p11.2;q21.2) translocation in papillary renal cell carcinoma fuses a novel gene PRCC to the TFE3 transcription factor gene. *Hum Molecular Genet* **5**: 1333–1338

Sonobe H, Manabe Y, Furihata M, Iwata J, Oka T, Ohtsuki Y, Mizobuchi H, Yamamoto K, Kumano O and Abe S (1992) Establishment and characterisation of a new human synovial sarcoma cell line HS-SY-II. *Lab Invest* **67**: 498–505

© 1999 Cancer Research Campaign

Thome M, Martinon F, Hofmann K, Rubio V, Steiner V, Schneider P, Mattmann C and Tschopp J (1999) Equine herpesvirus-2 E10 gene product, but not its cellular homologue, activates NF-κB transcription factor and c-Jun N-terminal kinase. *J Biol Chem* **274**: 9962–9968

Vogelstein B and Kinzler KW (1993) The multistep nature of cancer. *Trends Genet* **9**: 138–141

Willis TG, Jadayel DM, Du M-Q, Peng H, Perry A, Abdul-Rauf M, Price H, Karran L, Majekodunmi O, Wlodarska I, Pan L, Crook T, Hamoudi R, Isaacson P and Dyer MJS (1999) *BCL10* is involved in t(1;14)(p22;q32) of MALT B cell lymphoma and mutated in multiple tumor types. *Cell* **96**: 35–45

Yan M, Lee J, Schilbach S, Goddard A and Dixit V (1999) mE10, a novel caspase recruitment domain containing proapoptotic molecule. *J Biol Chem* **274** (15): 10287–10292

Yaoita S, Ohoi R, Hayashi Y, Kuwabara E and Watanabe T (1989) Establishment and characterisation of TN-2 cell line from a neuroblastoma of the left adrenal gland (in Japanese) *Hum Cell* **2**: 199

Zhang Q, Siebert R, Yan M, Hinzmann B, Cui X, Xue L, Rakestraw KM, Naeve CW, Beckmann G, Weisenberger DD, Sanger WG, Nowotny H, Vesely M, Callet-Bauchu E, Salles G, Dixit VM, Rosenthal A, Schlegelberger B and Morris SW (1999) Inactivating mutations and overexpression of BCL10, a caspase recruitment domain (CARD)-containing gene in MALT lymphoma with t(1;14)(p22;q32). *Nature Genet* **22**: 63–68

*© 1999 Cancer Research Campaign*

